# Approach to a cerebral hernia caused by an intratumoral hemorrhage of a cystic oligodendroglioma: a case report

**DOI:** 10.3389/fonc.2024.1295483

**Published:** 2024-04-03

**Authors:** Jiahua Zhou, Yingxi Wu, Huaizhou Qin, Shoujie Wang, Dayun Feng, Di Yang

**Affiliations:** ^1^ Department of Neurosurgery, Tangdu hospital, Air Force Medical University, Xi’an, Shaanxi, China; ^2^ Department of Radiology, Tangdu hospital, Air Force Medical University, Xi’an, Shaanxi, China

**Keywords:** cystic oligodendroglioma (COD), intratumoral hemorrhage (ITH), cerebral hernia, diagnosis, treatment

## Abstract

The incidence of cerebral herniation caused by intratumoral hemorrhage (ITH) in cystic oligodendroglioma (COD) is exceedingly rare. This study presents a case of cerebral herniation subsequent to cystic oligodendroglioma (COD) and sudden intratumoral hemorrhage. Following initial emergency treatment and evaluation, we successfully circumvented the solid component of the tumor and proceeded with cystic puncture and external drainage to prevent the incidence of brain herniation and mitigate the severity of associated symptoms. Based on preoperative examination results, the cystic glioma was successfully resected, and the patient experienced an uneventful recovery. According to the pathological findings, the oligodendroglioma was classified as World Health Organization (WHO) grade III. The treatment efficacy was comparable to cases of the same pathological grade, in which neither intratumoral hemorrhage nor cerebral hernia was observed.

## Introduction

The incidence rate of hemorrhage in patients with glioma is rather low, but most patients have high-grade gliomas, glioblastoma and gliosarcoma accounts for most cases (1). Some tumors are defined as WHO grade 3 and above; however, the symptoms of patients are usually serious, which necessitates emergency surgery or leads to death before surgical intervention (2). Oligodendroglioma accounts for 4-7% of all primary intracranial gliomas, which can be observed typically in young people and middle-aged adults (3). Meanwhile, the incidence of cystic oligodendroglioma (COD) is low (4). Particularly, COD with intratumoral hemorrhage (ITH) is extremely rare. This study reports the diagnosis and treatment of a rare case of cerebral hernia caused by intratumoral hemorrhage of cystic oligodendroglioma.

## Case report

### History and examination

**Figure 1:**
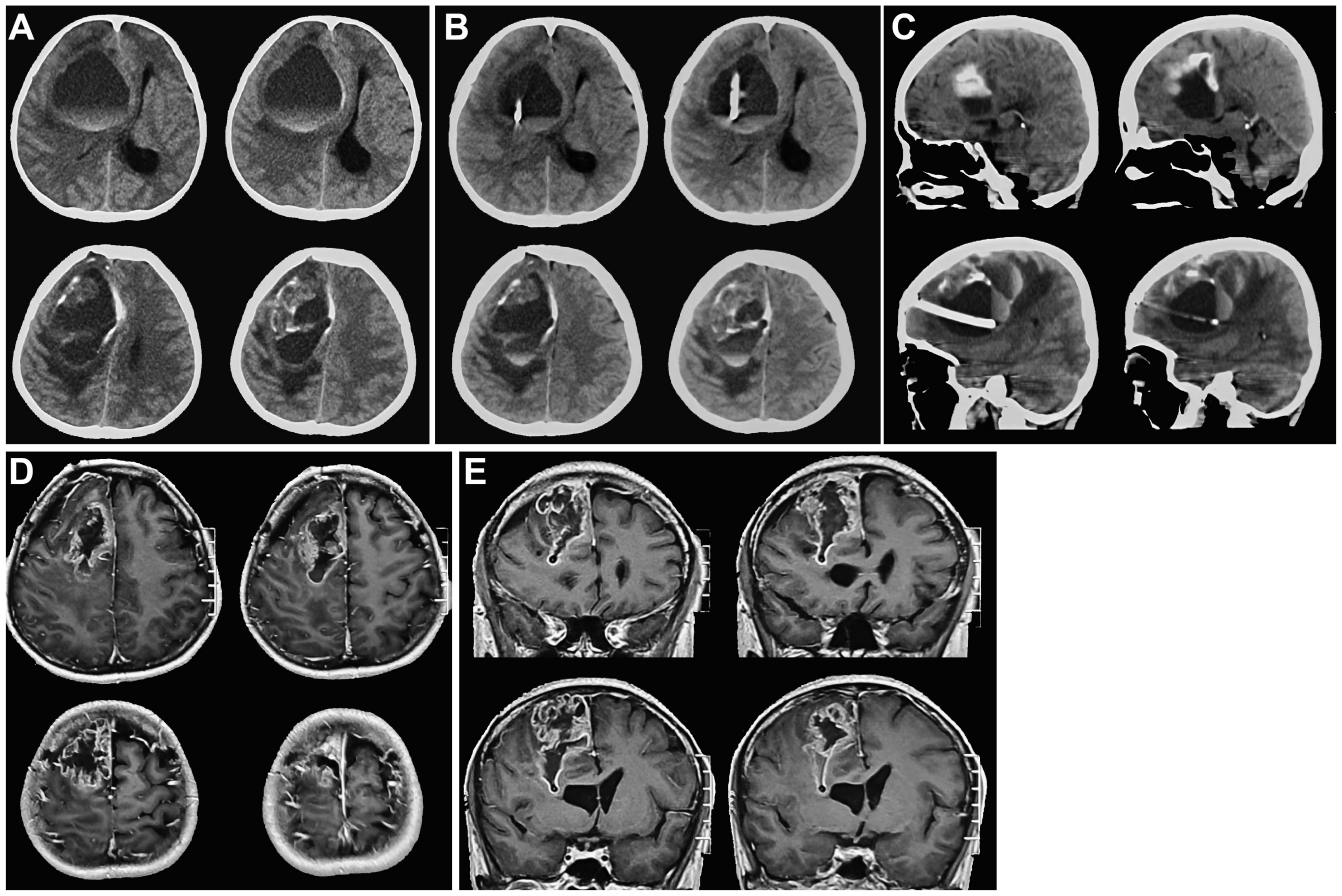
Cranial CT and MRI images before tumor resection. **(A)**. On admission, cranial CT showed a right frontal cystic lesion with fluid level. Cystic fluid was observed above blood. **(B, C)**. Axial and sagittal cranial CT images showed that the drainage tube was in the cyst, avoiding the solid component of the tumor after right frontal sac puncture. **(D, E)**. Axial and coronal cranial MRI images indicated that after the right frontal sac puncture, the sac fluid was discharged, and the solid component of the tumor was visible. The diagnosis was considered to be glioma.

Our patient was a female, aged 37, who was admitted to the medical facility on October 31, 2020, due to cephalalgia accompanied by left limb paresis persisting for 7 days. The symptoms subsequently exacerbated, leading to unconsciousness lasting for 4 hours. Cranial computed tomography (CT) scan (**Figure 1A**) revealed the presence of a cystic lesion in the right frontal lobe, measuring approximately 6.9×6.8×5.1cm, accompanied by hemorrhage. A fluid level was observed in the capsule. The upper portion of the observed specimen had a low density characterized by the presence of cystic fluid, whereas the lower portion displayed a high density primarily composed of blood. The right side of the midline was displaced approximately 2 cm, resulting in right lateral ventricle compression. After consultation with neurosurgeons and radiologists, the potential risk of hemorrhage subsequent to glioma was taken into consideration, and the patient was hospitalized in the neurosurgery ward. The patient did not have any notable medical history. The patient’s physical examination revealed a Glasgow Coma Scale (GCS) score of 6 (E1V1M4), suggesting mild coma. The left pupil had a diameter of approximately 3.0 mm, while the right pupil had a diameter of approximately 4.0 mm. Additionally, both pupils exhibited delayed light reflection.

### Preoperative assessment and treatment

**Figure 2:**
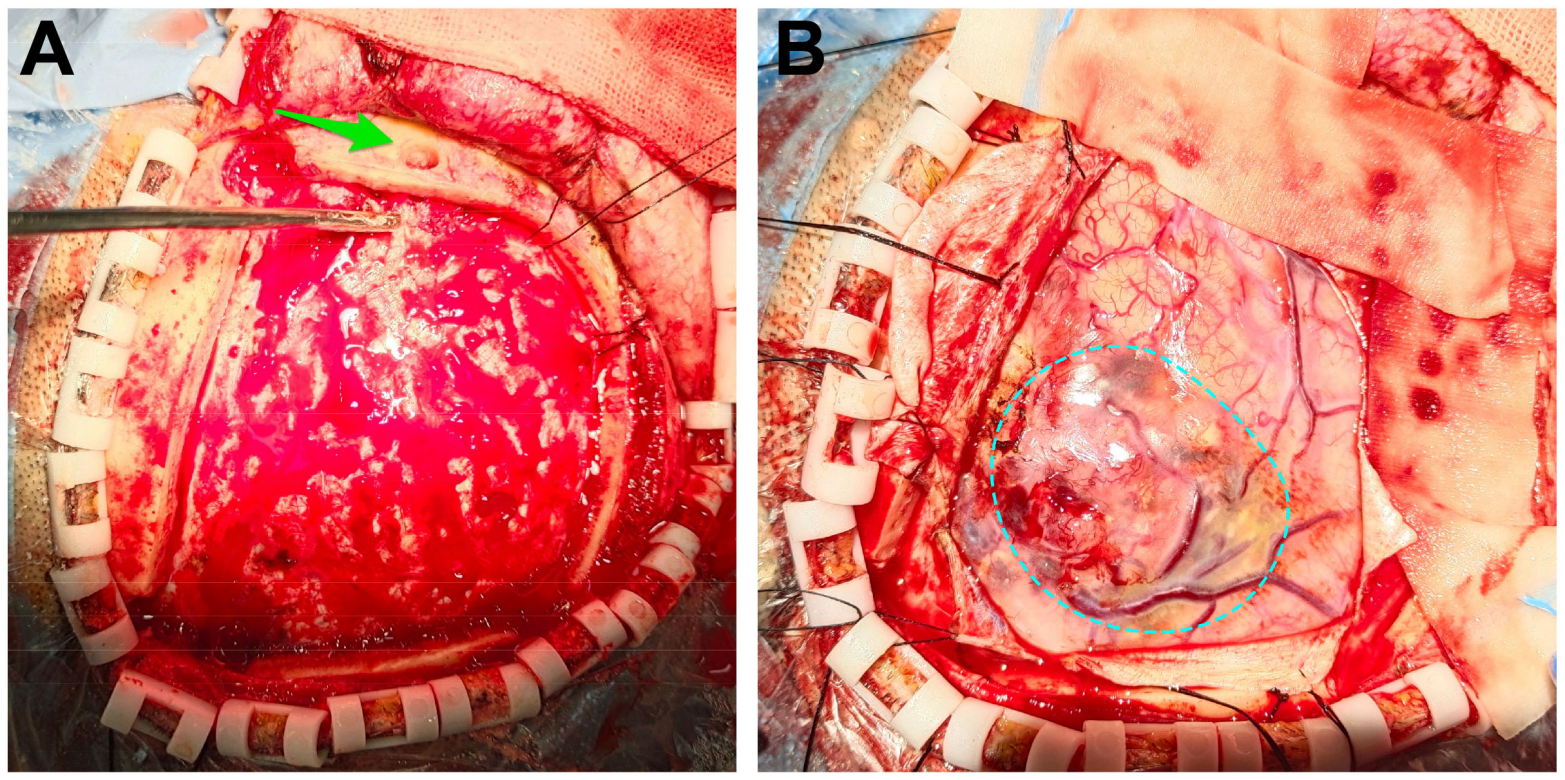
Intraoperative images. **(A)** We opened the flap and removed the bone flap. The puncture hole was seen (the green arrow). **(B)** After removing the dura mater, the tumor was seen (the dotted circle).

After admitting the patient to the neurosurgery ward, 250 ml of mannitol was infused. Nonetheless, the patients’ symptoms were not alleviated. We decided to perform frontal cystic fluid puncture and external drainage surgery to decrease high intracranial pressure and prevent cerebral hernia. Based on our clinical expertise and the available literature (5 6), it was determined that the transfrontal long axial puncture can yield superior outcomes compared to the transtemporal puncture, with a lower incidence rate of complication. Consequently, the transfrontal long axial puncture was chosen as the preferred puncture site. The puncture point for the procedure was 3 cm from the forehead eyebrow arch and 3 cm from the midline aperture. This point is parallel to the sagittal plane and is directed toward the tumor cavity. After reaching a depth of approximately 4 cm, a liquid with a yellow-red hue was released. Then, we extracted the pierce needle core and advanced forward for 2 cm. The drainage tube exhibited no signs of obstruction. After fixing the drainage tube, the surgical procedure was successfully finished. Reevaluation cranial CT scan revealed that the drainage tube was appropriately positioned into the tumor cavity, avoiding damage to the solid component of the tumor. The cyst fluid was slowly released to avoid rebleeding. Additionally, no evidence of fresh hemorrhage was observed (**Figures 1B, C**). After 12 hours, the patient regained consciousness. Cranial magnetic resonance imaging (MRI) revealed a glioma in the right frontal region (**Figures 1D, E**). The fluid within the capsule was effectively evacuated, and the midline of the brain was located approximately at its central position. The surgical procedure for resecting the right frontal lobe tumor was conducted on November 3. After administering anesthetics, the drainage tube of the tumor cavity was successfully extracted. Subsequently, the skull frame was positioned. During the surgical procedure, a frontal coronal flap was utilized to create a bone window measuring approximately 5×6 cm (**Figures 2A, B**). The dura mater was incised, providing access to the tumor, which was identified using ultrasound guidance. Subsequently, the tumor was completely excised using a microscope. Moreover, the wall of the capsule was entirely eliminated. The dura mater was sutured, the drainage tube was positioned outside the dura mater, the bone flap was repositioned, and the surgical procedure was completed.

### Postoperative course

**Figure 3:**
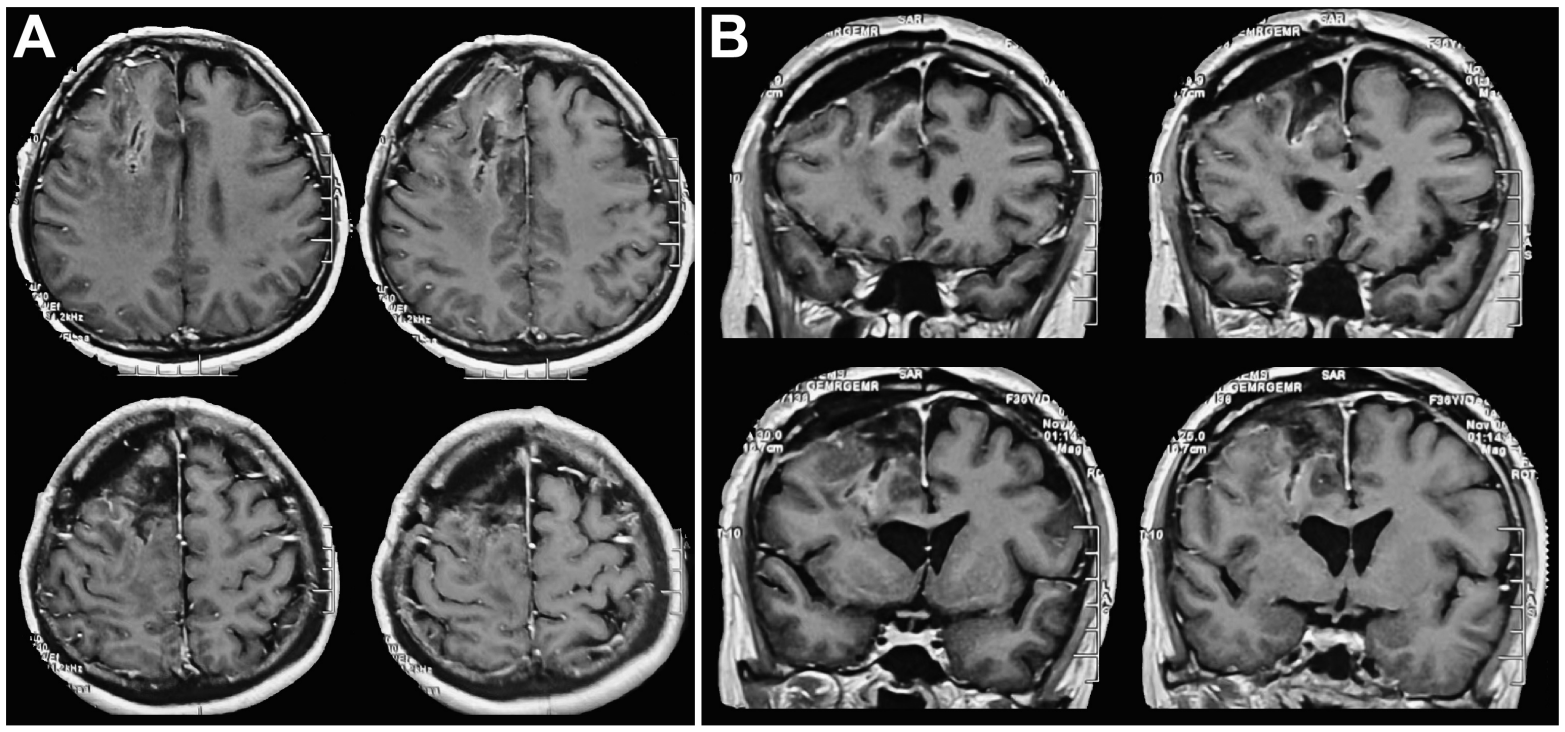
Postoperative head MRI findings. **(A)** Postoperative axial cranial MRI indicated that the tumor was completely removed. **(B)** Postoperative coronal cranial MRI indicated that the tumor was completely removed, the midline was in the center, and ventricular compression was relieved.

**Figure 4:**
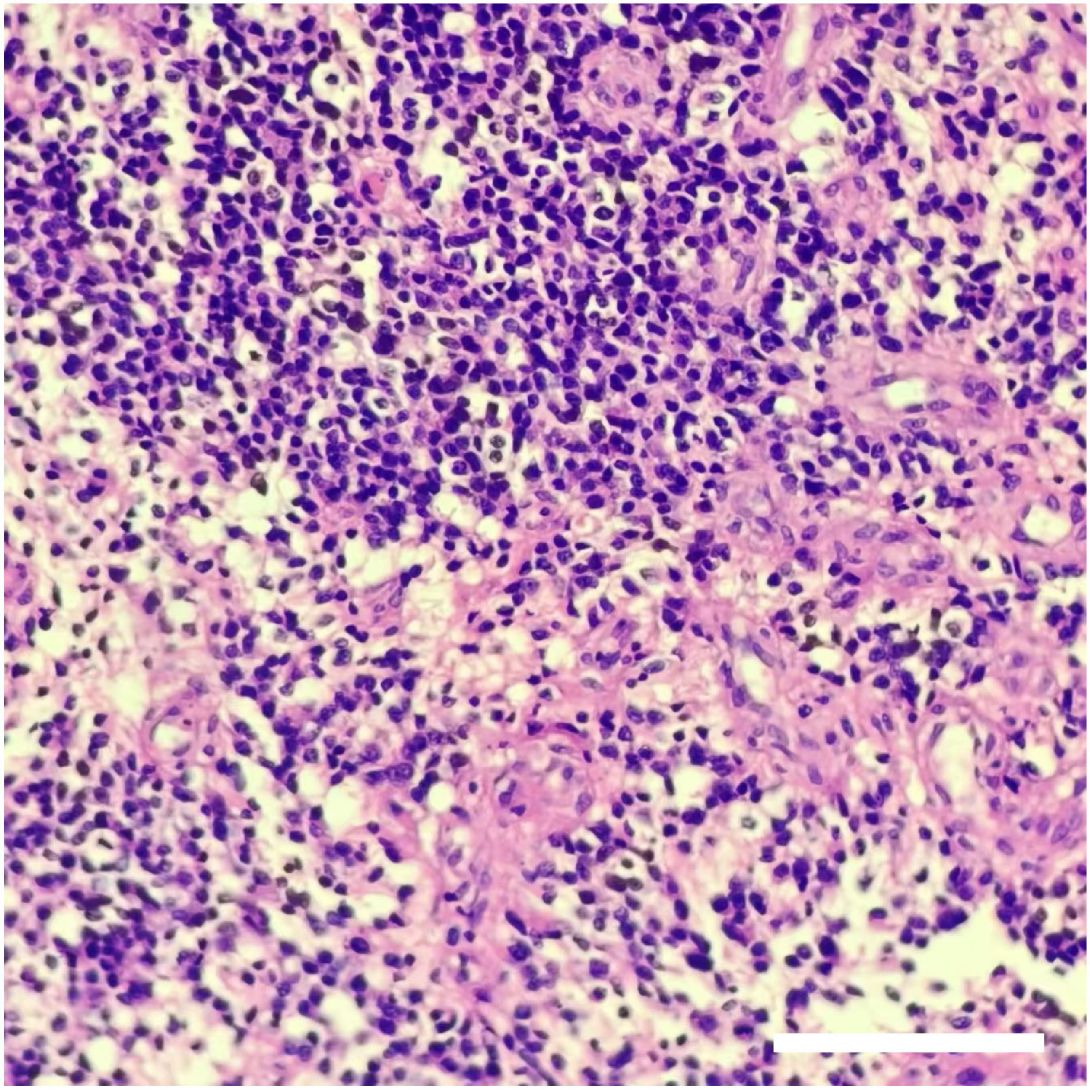
The result of pathological assessments. H&E staining showed that the density of tumor cells increased, the cells were round or oval, and perinuclear halos and nucleoli were visible. Local microvascular hyperplasia of the tumor. The pathological results indicated that the tumor was oligodendroglioma (WHO grade III). Scale bar: 200 μm.

The patient received symptomatic care, including prophylaxis for infection and epilepsy. After completing treatment, the patient was discharged without complications. Notably, her language and limb skills showed a significant recovery. Reevaluation of the cerebral magnetic resonance imaging (MRI) revealed that the tumor was completely resected (**Figures 3A, B**). Pathological analysis revealed grade 3 oligodendroglioma (**Figure 4**). Additionally, the tumor was identified as an IDH-mutant oligodendroglioma with 1p/19q co-deletion.

### Postoperative treatment and follow-up for COD

Following the surgical procedure, a single postoperative cycle of concomitant radiation and chemotherapy was administered, followed by six cycles of temozolomide single-drug chemotherapy. Over 34 months of follow-up, the patient generally exhibited a favorable health condition, evidenced by a Karnofsky score of 100 and the absence of any signs of tumor recurrence.

## Discussion

Currently, there exists a limited number of reports pertaining to cystic oligodendroglioma, predominantly consisting of case reports (5–10). Nevertheless, there are few cases of cerebral hernia caused by the combination of cystic oligodendroglioma and intratumoral hemorrhage. It has been reported that patients with large cystic metastatic tumors and cystic glioblastomas may benefit from cystic aspiration before craniotomy. Cyst aspiration leads to the expansion of the compressed brain tissue between the tumor periphery and important structure, which makes it suitable for optimal and safe resection (4, 11). In patients with cystic glioma presenting with intratumoral hemorrhage and brain hernia, prompt intervention in the form of cystic fluid puncture and external drainage is imperative to alleviate symptoms. The subsequent surgical procedure is rather straightforward. The co-incidence of gliomas and bleeding frequently and suddenly manifests as a severe clinical state (12). Individuals diagnosed with cerebral hernias need prompt medical intervention when conservative treatment is insufficient. The challenging nature of the critical condition prevents the completion of a comprehensive and detailed examination, thus increasing the potential for misdiagnosis and mistreatment (13). In this particular scenario, it is recommended to initiate the procedure by performing the puncture and conducting subsequent external drainage of the cystic fluid. Following the discharge of cystic fluid, the intracranial pressure becomes relatively normal, improving the patient’s level of consciousness and even leading to full wakefulness. This allows for a valuable window of opportunity to undertake necessary preoperative measures. Once patients can participate, standard cranial MRI and other diagnostic tests are performed to confirm the diagnosis. Tumor resection can be conducted following a thorough preparation. The success rate of the procedure is higher, improving the therapeutic efficacy. Neglecting the hemorrhage within the glioma and solely relying on craniotomy for hematoma removal may result in inadequate tumor resection or incomplete tumor removal, compromising the efficacy of treatment.

The incidence of intratumoral hemorrhage in gliomas is predominantly attributed to factors such as malignant behavior, rapid proliferation, intratumoral necrosis, or angiogenesis (14). A comprehensive three-dimensional understanding is needed to perform a puncture procedure. This enables the precise insertion of the needle into the healthy brain tissue, thereby preventing contact with the solid components of the tumor and minimizing the risk of subsequent hemorrhage. Such meticulous puncturing techniques are crucial to prevent the exacerbation of patients’ condition. When hemorrhage and subsequent brain herniation persist following penetration of the tumor, an urgent craniotomy procedure becomes imperative to extract both the hematoma and the tumor. The glioma of our patient was in the frontal lobe, and the cystic component was adjacent to the right lateral ventricle, compressing the right lateral ventricle and leading to a notable leftward shift of the midline. Based on our clinical expertise and the pertinent literature (15, 16), it has been determined that the efficacy of long frontal axis puncture surpasses that of temporal puncture, with a reduced incidence of complications. Consequently, we selected the long frontal axis puncture as our preferred puncture point. This puncture method takes the forehead eyebrow arch 3 cm and the opening 3 cm on the left side of the midline as the puncture point, points to the cystic cavity, successfully avoids the solid component of the tumor, and releases the yellow-red cystic fluid after entering the tumor cavity. This approach has been demonstrated to be both secure and efficient (15, 16). After the discharge of cystic fluid, the symptoms associated with elevated intracranial pressure were alleviated, restoring the patient’s consciousness. This improvement in consciousness facilitated the opportunity for subsequent assessments and interventions.

COD is resected the same as conventional cystic gliomas. Gliomas with intratumoral hemorrhage are generally regarded as high-grade gliomas (17). Using a microscope, experienced neurosurgeons can distinguish the tumor tissue from healthy brain tissue. Before excising the tumor and after incising the dura mater, it is helpful to perform ultrasonic localization, as this technique precisely demarcates the tumor (18, 19). In conjunction with microscopic observations, total excision of the solid tumor tissue and subsequent removal of the entire capsule wall can prevent tumor recurrence. Incomplete excision of the tumor increases the risk of recurrence, proliferation, and hemorrhage. In this particular scenario, the surgical outcome is suboptimal (20). When the tumor is located in close proximity to a nonfunctional region, it may be feasible to increase the resection scope. This approach can potentially delay tumor recurrence. Based on the findings of the pathological examination, postoperative care includes routine follow-up and regular monitoring. COD recurrence necessitates another round of resection.

## Conclusions

The incidence of cerebral hernia due to COD, in conjunction with intratumoral hemorrhage (ITH), is extremely rare, necessitating prompt medical intervention. Tumor cavity puncture can be performed, and cystic fluid can be slowly released under the premise of avoiding the tumor entity. This treatment can reduce intracranial pressure, alleviate patients’ condition, and provide enough time for conservative treatment and necessary examinations, such as cranial plain and enhanced MRI. Surgical procedures can be performed once preoperative preparations have been completed. This is the first reported effective diagnosis and management of such a case. In patients with favorable post-puncture outcomes, total tumor removal can be achieved through surgical intervention. Following surgical intervention, a comprehensive treatment approach, including radiotherapy and chemotherapy, may be employed, guided by pathological findings. This therapeutic strategy can yield a prognosis that is equivalent to that of patients with COD without cerebral hernia subsequent to intratumoral hemorrhage.

## Data availability statement

The datasets presented in this article are not readily available because no restriction. Requests to access the datasets should be directed to zhoujiahuatd@163.com; yangdimed@163.com.

## Ethics statement

Written informed consent was obtained from the individual(s) for the publication of any potentially identifiable images or data included in this article. Written informed consent was obtained from the participant/patient(s) for the publication of this case report.

## Author contributions

JZ: Methodology, Supervision, Writing – original draft, Writing – review & editing. YW: Conceptualization, Investigation, Writing – review & editing. HQ: Data curation, Investigation, Methodology, Writing – review & editing. SW: Investigation, Methodology, Writing – review & editing. DF: Conceptualization, Investigation, Methodology, Project administration, Writing – review & editing. DY: Conceptualization, Methodology, Investigation, Writing – review & editing.
